# Nitrogen and carbon concentrations and stable isotope ratios: Data from a ^15^N tracer study in short-form *Spartina alterniflora* and *Distichlis spicata*

**DOI:** 10.1016/j.dib.2018.09.133

**Published:** 2018-10-10

**Authors:** Troy D. Hill, Nathalie R. Sommer, Caroline R. Kanaskie, Emily A. Santos, Autumn J. Oczkowski

**Affiliations:** a United States Environmental Protection Agency, Office of Research and Development, Narragansett, RI, United States; b Yale University, School of Forestry and Environmental Studies, New Haven, CT, United States; c University of New Hampshire, Department of Natural Resources and the Environment, Durham, NH, United States; d Humboldt State University, College of Natural Resources and Sciences, Arcata, CA, United States

## Abstract

We present four datasets that provide information on primary production, nitrogen (N) uptake and allocation in two salt marsh grasses, short-form *Spartina alterniflora* and *Distichlis spicata*. These four datasets were generated during a month-long stable isotope (^15^N) tracer study described in the companion manuscript (Hill et al., 2018). They include an allometry dataset containing mass and height data for individual plants harvested from Colt State Park, Bristol, Rhode Island and used to nondestructively estimate plant masses. A second dataset contains weekly stem height measurements collected over the course of the ^15^N tracer study. Also included are high resolution data from 49 vegetated compartments (leaves, stems, fine/coarse roots, rhizomes) and bulk sediment depth intervals, reporting the mass, carbon and N concentrations, and stable isotope ratios measured following the harvest of cores over time. Additionally, we provide a complementary dataset with estimates of microbial removal from potential and ambient denitrification enzyme assays. These data, along with source code used in their analysis, are compiled in the NitrogenUptake2016 R package available from the Comprehensive R Archive Network.

**Specifications table**

**Table:** 

Subject area	Chemistry, Biology, Ecology
More specific subject area	Biogeochemistry, plant ecology
Type of data	R package, figures
How data were acquired	Nutrient concentrations and stable isotope ratios: Elementar Vario Micro elemental analyzer connected to a continuous flow Isoprime 100 isotope ratio mass spectrometer
	Denitrification enzyme assays: N_2_O concentrations were measured on a Shimadzu GC2014 gas chromatographer with an electron capture detector
	Stem heights: ruler (±0.1 cm)
	Biomass: electronic scale (±0.001 g)
Data format	Raw and analyzed
Experimental factors	Fifteen salt marsh sediment cores were collected from each of two vegetation types (short form *Spartina alterniflora* and *Distichlis spicata*). Cores were transferred to and grown in a laboratory greenhouse and deconstructed over four weeks following a single ^15^N tracer addition.
Experimental features	Three cores from each vegetation type were physically deconstructed at weekly intervals to measure ^15^N accumulation in a range of vegetative and sedimentary compartments. Additional data are provided describing the mass-height allometry of aboveground biomass, the dataset of weekly stem height measurements used to estimate growth rates, and results of sediment denitrification enzyme assays.
Data source location	Cores and allometry samples were collected from Colt State Park, Bristol, Rhode Island, USA (Latitude/Longitude: 41.6857, -71.2885). Greenhouse incubations conducted at USEPA Atlantic Ecology Division, Narragansett, Rhode Island, USA.
Data accessibility	Data are publicly available as an R package archived by the Comprehensive R Archive Network (https://cran.r-project.org/package=NitrogenUptake2016).

**Value of the data**

• Stable isotope, nutrient concentration, and biomass data provide weekly snapshots of ^15^N tracer uptake and allocation to a suite of above- and belowground tissues.
• Data allow investigation of nitrogen budgets and cycling in salt marsh ecosystems.
• Growing season mass-height allometry data for Rhode Island, USA can be used in salt marsh studies from the northeast, compared to other geographic areas, and used in meta-analyses.
• Denitrification enzyme data can be used in syntheses or meta-analyses.

## 1 Data

### 1.1 Experimental context

Salt marshes are exceptionally productive ecosystems [2]. High rates of primary production lead to substantial nutrient uptake by salt marsh plants [3], and biogeochemically active marsh sediments intercept nitrogen (N) dissolved in coastal waters [4], [5], [6]. To examine plant N uptake and possible artifacts associated with time scales of stable isotope tracer studies, we collected salt marsh cores (“mesocosms” hereafter) from monoculture zones of short-form *Spartina alterniflora* and *Distichlis spicata* and amended them with ^15^N. Nitrogen uptake and allocation were examined over the subsequent four weeks at high temporal and spatial resolution.

This work generated several datasets described in this paper and contained in the associated R package, NitrogenUptake2016. The R package can be installed and loaded by running the following commands in R:

install.packages("NitrogenUptake2016")

library("NitrogenUptake2016")

The four datasets are automatically loaded into the R session and their documentation pages are accessible in the R console using the following commands:

?allometry

?stemHeights

?CN_mass_data

?dea

### 1.2 Mass-height allometry

Live plants were collected from Colt State Park (Bristol, Rhode Island, USA) in May, June, and July 2016. On each sampling date, three 25 cm × 25 cm quadrats were collected from monoculture areas of short-form *S. alterniflora* and *D. spicata*. Stems were cut at the sediment surface, total plant height was measured to the nearest 0.1 cm, and a representative subsample of plants were individually dried to constant weight at 50 °C. The “allometry” dataset includes 95 mass/height observations (units: grams and centimeters) from *D. spicata* and 75 from short-form *S. alterniflora*.

### 1.3 Stem heights

All live plants in each of the 24 mesocosms were tagged and the height from the base to the tallest feature was measured to the nearest 0.1 cm on 22 June, 29 June, 6 July, 13 July, and 20 July 2016. Additional height measurements were recorded as mesocosms were harvested incrementally over a four-week period. These data are included in the “stemHeights” dataset. New plants were tagged throughout the experiment, resulting in 3315 height measurements from 839 unique plants.

### 1.4 Mass, nutrient, and stable isotope concentrations

Mesocosm harvests occurred on 1 July, 8 July, 15 July, and 22 July 2016. During harvest, material in the mesocosms was separated into as many as 49 distinct vegetated compartments (leaves divided by node, stems, fine/coarse roots, rhizomes) and bulk sediment depth intervals. Aboveground and belowground compartments are described in the Experimental Methods section, below. In addition to the dry mass (50 °C) of each compartment in each mesocosm, this dataset contains nitrogen and carbon concentrations and stable isotope ratios.

The “CN_mass_data” dataset contains 1192 observations reporting the mass (g) of each compartment recovered from each mesocosm, the compartment׳s volume (cm^3^; for belowground samples), carbon and nitrogen content (g·g^-1^), and stable carbon and nitrogen isotope ratios (per mil deviations from Vienna Pee Dee Belemnite (VPDB) and Air, respectively).

### 1.5 Denitrification enzyme assays

The data are derived from two sets of acetylene inhibition assays conducted using bulk sediment from 0 to 5 cm depth intervals of each of the six mesocosms harvested on 9 July 2016. The two assays measured denitrification enzyme activity (DEA) potential and “*in vitro*” or ambient denitrification. Both rates are provided on a per-gram basis (units: nmol N_2_O · g dry mass^−1^ · h^−1^) in the “dea” dataset.

## 2 Experimental design, materials and methods

### 2.1 Experimental context

Thirty salt marsh sediment mesocosms (10 cm × 35 cm PVC cores; 15 from monocultures of each species) were collected from the field, and three from each vegetation type were immediately harvested to represent time-zero conditions. The remaining 24 mesocosms were grown in a tidal tank in an outdoor greenhouse, and a single dose of 1.95 mg ^15^N was added to each mesocosm on 24 June 2016. Mesocosms were then harvested at weekly intervals over the subsequent four weeks.

### 2.2 Mass-height allometry

Live plants collected from Colt State Park, Bristol, RI, USA were used to establish allometric relationships between plant׳s height and mass. Masses were modeled as a function of height, with species-specific allometry models parameterized using Box-Cox power transformations of biomass (*λ*). Masses of plants growing in the mesocosms were estimated from weekly plant height measurements using species-specific allometry equations of the form mass = (height · *a* + *b*)^1/*λ*^. The R code used to construct these models from raw mass and height data is included in the “JEMBE” vignette in the R package “NitrogenUptake2016,” accessible by running the following commands in R:

vignette(topic = "JEMBE", package = "NitrogenUptake2016")

### 2.3 Stem heights

Using source code provided in the package vignette, the species-specific allometry models shown in Table 1 from Hill et al. [1] were applied to stem heights collected over the course of the experiment to estimate biomass. Stem-level masses were summed by mesocosm for each measurement interval, and net primary production was calculated as the sum of positive live biomass increments at the mesocosm-scale [7], [8].

**Fig. 1:**
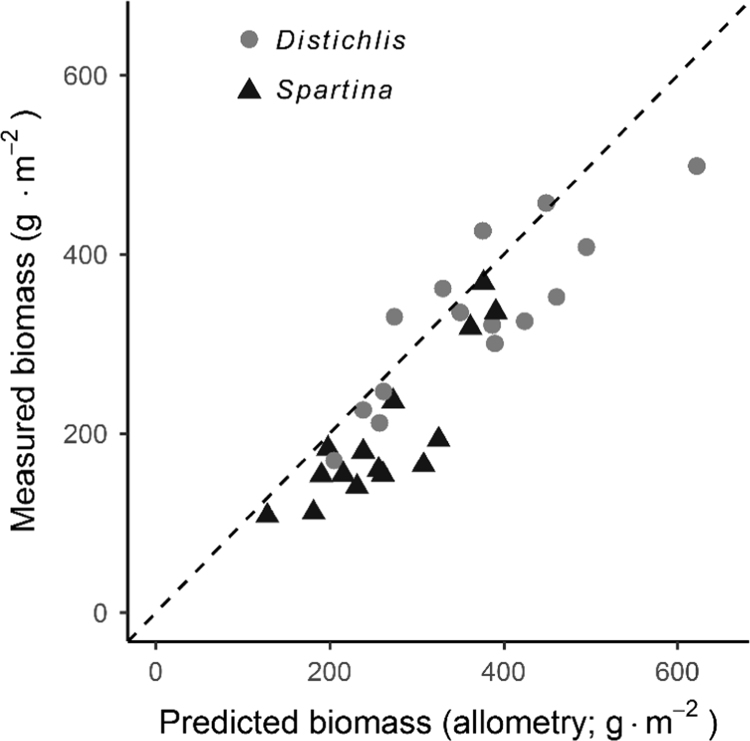
Relationship between allometry-based biomass estimates and biomass measured at harvest for each mesocosm (*R*^*2*^ = 0.80, *y* = 0.87x, *P* < 0.001), with dashed 1:1 line shown.

Mesocosm harvests provided an opportunity to examine the applicability of these models to plants grown in our greenhouse experiment. Aboveground biomass measured during mesocosm harvest was compared with predicted biomass; the sum of stem-level masses estimated from allometry models. These data are shown in Fig. 1 (*R*^*2*^ = 0.80, *y* = 0.87x, *P* < 0.001).

### 2.4 Mass, nutrient, and stable isotope concentrations

At collection (21 June 2016) and at weekly intervals following tracer addition, cohorts of three randomly selected mesocosms from each species was harvested for analysis of ^15^N accumulation in above- and belowground compartments. Aboveground biomass was clipped at the sediment surface, rinsed with deionized water and separated into live and standing dead plants. Live plants were further separated into stems, dead leaves, and live leaves. Live leaves were separated and numbered by position relative to the top of the plant. These node-level data are included in the provided dataset, although they were combined into a single leaf pool during data analysis [1].

After aboveground biomass was sampled, peat was extruded from the coring tube and sectioned into six depth intervals: 0–2 cm, 2–5 cm, 5–10 cm, 10–15 cm, 15–20 cm, and 20–30 cm. Surface litter and algal mat layers were separated when present. Each sediment depth interval was subsampled for belowground biomass and bulk density. One quarter of each depth interval volume was dried to constant weight at 50 °C, and bulk density calculated as the dry mass divided by the sample volume. One quarter of each depth interval was archived in a freezer, and the remaining half of each depth interval was used for separation of belowground biomass. Within one week of harvest, belowground biomass was rinsed clean of sediment using deionized water and separated into live rhizomes, live coarse roots (>1 mm), live fine roots (≤ 1 mm), and dead biomass. Live biomass was distinguished from dead by its white color and turgid structure. All samples were dried to constant weight at 50 °C, ground using a Wiley mill and a size 40 screen, and stored in acid-washed scintillation vials. Samples containing ^15^N tracer samples were ground on a separate dedicated mill to prevent contamination.

Nitrogen and carbon concentrations and stable isotope ratios were measured using an Elementar Vario Micro elemental analyzer connected to a continuous flow Isoprime 100 isotope ratio mass spectrometer. Check standards, blanks, and replicated samples were run every ten samples. Replicate analyses of isotopic standard reference materials USGS 40 (δ^13^C = −26.39‰; δ^15^N = −4.52‰) and USGS 41 (δ^13^C = 37.63‰; δ^15^N = 47.57‰) were used to normalize isotopic values of working standards to the Air (δ^15^N) and VPDB (δ^13^C) scales [9]. Average recoveries for standard reference materials were ±1.1% for δ^15^N, ±0.4% for total N, ±0.03% for δ^13^C, and ±0.05% for total C. Coefficients of variation on replicate samples averaged 8.9% for δ^15^N, 5.9% for total N, −0.5% for δ^13^C, and 3.6% for total C.

**Fig. 2:**
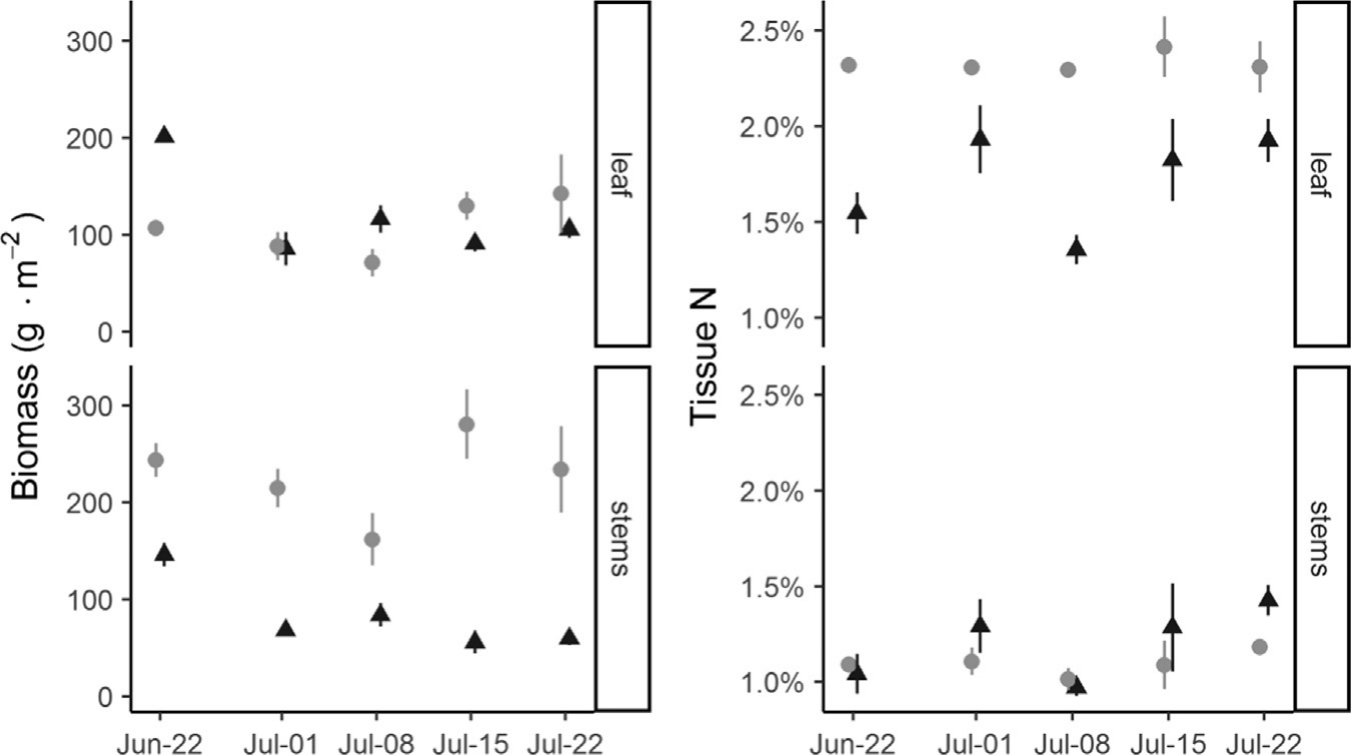
Mean (±SE; *n* = 3) leaf and stem biomass (left side) and N content (right side) at each harvest. *Distichlis* shown as gray points, *Spartina* as black triangles.

**Fig. 3:**
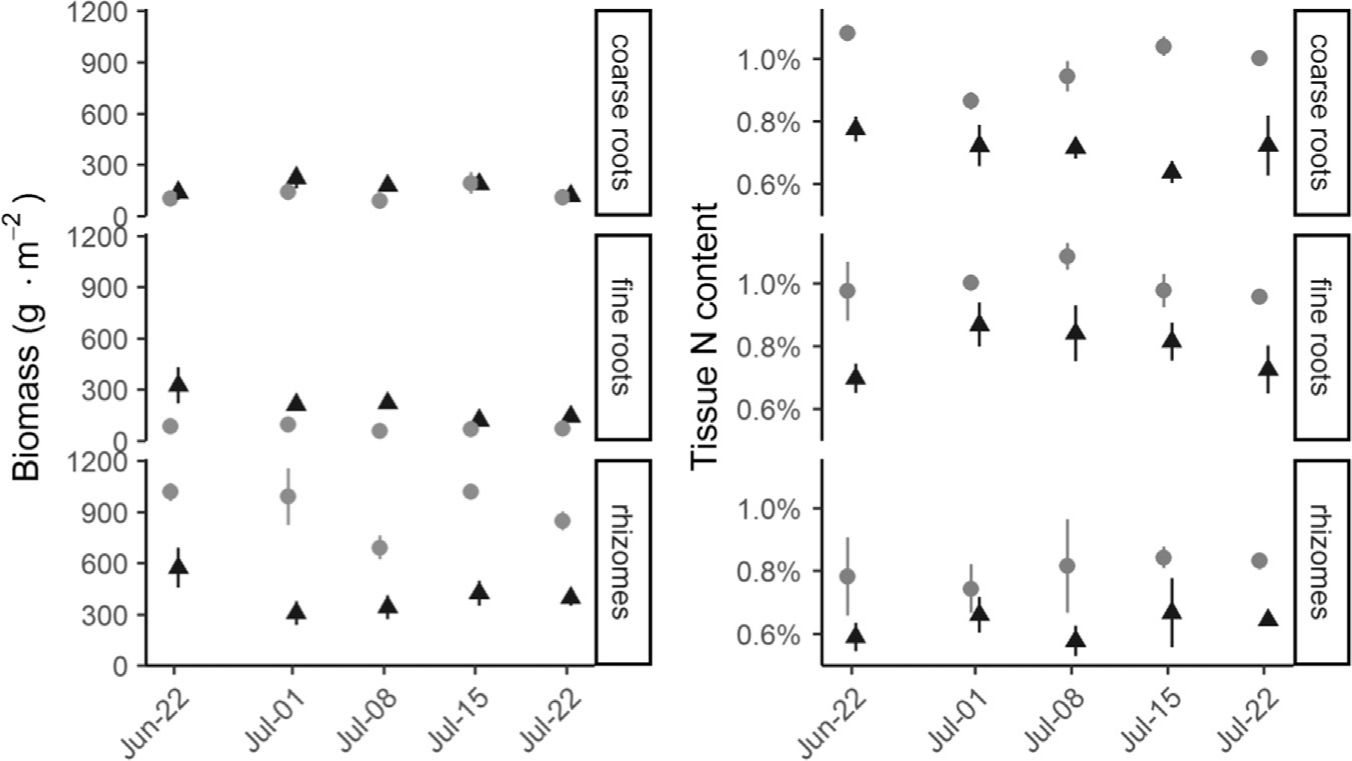
Mean (±SE; *n* = 3) belowground biomass (left side) and N content (right side) at each harvest. *Distichlis* shown as gray points, *Spartina* as black triangles.

These compartment-level data provide a time series of biomass and tissue N in aboveground (Fig. 2) and belowground (Fig. 3) tissues.

### 2.5 Denitrification enzyme assays

DEA potential was measured by incubating 6.5 g of sediment in 70 mL jars sealed with rubber septa. Sample containers were amended with 12 mL of nutrient-amended filtered seawater (7 mmol N·L^−1^ as KNO_3_; 17 mmol C·L^−1^ as d-glucose; and 6 mmol P·L^−1^ as KH_2_PO_4_) with 0.125 g·L^−1^ chloramphenicol added as a microbial inhibitor. Containers were alternately evacuated and flushed with N_2_ three times, and acetylene (10% of headspace volume) was added to block reduction of N_2_O to N_2_. *In vitro* denitrification was measured in nearly identical acetylene inhibition assays. The only difference was the use of 12 mL of un-amended filtered seawater rather than a nutrient solution [10].

In both assays, N_2_O accumulation in the jars was measured at 30 min intervals for two hours. During sampling, 5 mL of sample headspace and 10 mL of N_2_ were transferred to evacuated 12 mL exetainers (Labco, UK). Sampled headspace was replaced with a 10% mixture of acetylene in N_2_. Nitrous oxide concentrations were analyzed by gas chromatography (GC; Shimadzu GC2014) using an electron capture detector (ECD). The GC column oven temperature was 75 °C, the ECD temperature was 325 °C, the carrier gas was helium and makeup gas was a 5% solution of methane in argon. The potential and *in vitro* DEA data provided here reflect the slope of the line of best fit for dilution-corrected N_2_O concentrations in the jar headspace. At least three points were used for each rate estimate, and all relationships had *R*^*2*^ ≥ 0.92 (mean *R*^*2*^ = 0.97). A laboratory blank using DEA solution and no added sediment yielded zero flux (slope = 0.0; *R*^*2*^ = 0.0), so no blank correction was applied.
