# Supplementary material for: Nitrogen and carbon concentrations and stable isotope ratios: Data from a 15N tracer study in short-form Spartina alterniflora and Distichlis spicata
Source: Data Brief. 2018 Oct 10;21:466–72. doi: 10.1016/j.dib.2018.09.133 (PMC6198123; doi:10.1016/j.dib.2018.09.133)
Supplement: Supplementary file 1 — Supplementary material [file mmc1.docx]

Author Conflict of Interest Declaration

We wish to confirm that there are no known conflicts of interest associated with this

publication and there has been no significant financial support for this work that could have

influenced its outcome.

We confirm that the manuscript has been read and approved by all named authors and that

there are no other persons who satisfied the criteria for authorship but are not listed. We

further confirm that the order of authors listed in the manuscript has been approved by all of

us.

We confirm that we have given due consideration to the protection of intellectual property

associated with this work and that there are no impediments to publication, including the

timing of publication, with respect to intellectual property. In so doing we confirm that we

have followed the regulations of our institutions concerning intellectual property.

We understand that the Corresponding Author is the sole contact for the Editorial process

(including Editorial Manager and direct communications with the office). He/she is

responsible for communicating with the other authors about progress, submissions of

revisions and final approval of proofs. We confirm that we have provided a current, correct

email address which is accessible by the Corresponding Author and which has been

configured to accept email from [Hill.Troy@gmail.com](mailto:Hill.Troy@gmail.com).

Signed by all authors as follows:

Troy Hill:
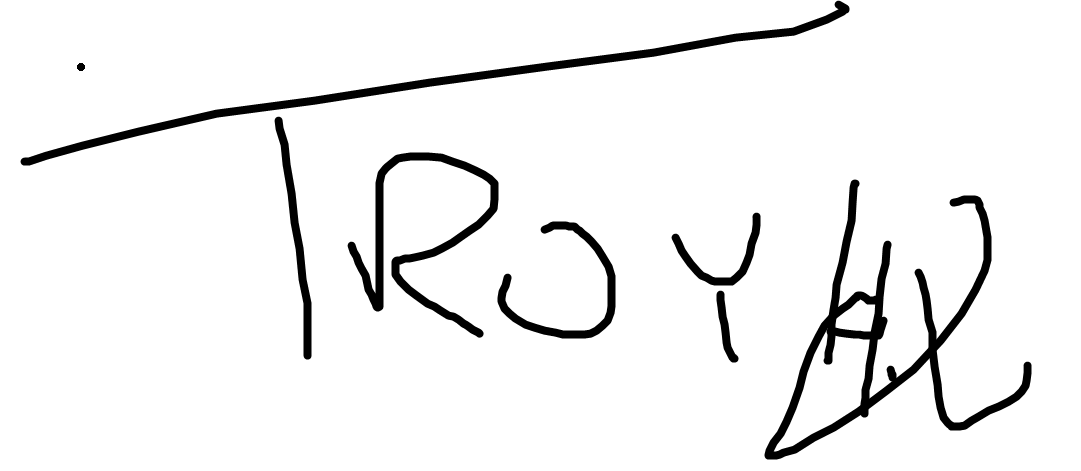
, 22 August 2018

Nathalie Sommer:
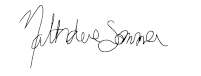
, 22 August 2018

Caroline Kanaskie:
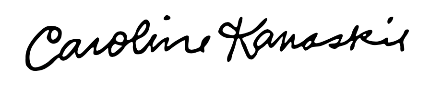
, 22 August 2018

Emily Santos:
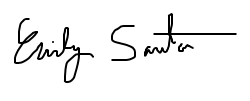
, 22 August 2018

Autumn Oczkowski:
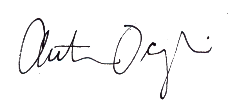
, 22 August 2018
